# Spectral Characterization of a Prototype SFA Camera for Joint Visible and NIR Acquisition

**DOI:** 10.3390/s16070993

**Published:** 2016-06-28

**Authors:** Jean-Baptiste Thomas, Pierre-Jean Lapray, Pierre Gouton, Cédric Clerc

**Affiliations:** LE2I UMR CNRS 6306, Université de Bourgogne, Dijon 21000, France; plapray@gmail.com (P.-J.L.); pgouton@u-bourgogne.fr (P.G.); cedric.clerc@u-bourgogne.fr (C.C.)

**Keywords:** multispectral imaging, spectral filter array, sensors

## Abstract

Multispectral acquisition improves machine vision since it permits capturing more information on object surface properties than color imaging. The concept of spectral filter arrays has been developed recently and allows multispectral single shot acquisition with a compact camera design. Due to filter manufacturing difficulties, there was, up to recently, no system available for a large span of spectrum, i.e., visible and Near Infra-Red acquisition. This article presents the achievement of a prototype of camera that captures seven visible and one near infra-red bands on the same sensor chip. A calibration is proposed to characterize the sensor, and images are captured. Data are provided as supplementary material for further analysis and simulations. This opens a new range of applications in security, robotics, automotive and medical fields.

## 1. Introduction

While MultiSpectral Imaging (MSI) is now a solution commonly considered for several problems and wide range applications, i.e., medical imaging, security, automotive, earth, food and cultural heritage [1,2,3,4], there is still the need to develop a compact and affordable solution to generalize its use.

MSI is defined as the mean to obtain a multispectral image *I*. Such multispectral image is formed of $I_{k}$ layers, that correspond to the *k* spectral sensitivities defined for a specific MSI system. The case of color imaging is a specific subset of MSI, where k=3.

Although there has been extended research on how to acquire multispectral images [5], the complexity and need for tunability of such MSI system limits its use to some specialized areas and often requires experts to handle them.

In digital color imaging, the concept of Color Filter Array (CFA) has been exploited since the 1970s and the invention of the Bayer pattern [6]. Indeed, at the expense of spatial resolution, one can increase the spectral resolution of an imaging device. The optical and spectral characteristics of such a setup have been extensively studied during the last decades [7,8,9,10,11,12,13]. Also, the demosaicing of such sensors has been investigated [14], and in general, the CFA camera processing pipeline is well understood [15].

Recent studies considered the problem of extending and generalizing the CFA concept to Spectral Filter Array (SFA), where an arbitrary spatio-spectral sampling of the image captured may be performed by the sensor, even beyond visible limits. A comprehensive review of single shot MSI technologies may be found in the work of Hagen and Kudenov [16]; and an exhaustive review of SFA sensors can be read in the work of Lapray et al. [17].

Much effort has been put into the realisation of spectral filters for SFA cameras, and the Fabry-Perot interferometric system, as realized by nano-etching, seems to be the dominant process when it comes to the realisation of matrices of pixels of small dimension [18,19]. However, despite the simulation made at LETI [18], no realization of filters based on Fabry-Perot interferometer that cover the visible and Near Infra-Red (NIR) range has been made in practice. An attempt by Lapray et al. combined nano-etching on one side of the substrate to handle the visible part of the spectrum and thin layer deposition on the other side to handle the NIR part of the spectrum. Beside, a great improvement has been achieved on spectral filters based on nano-tube, and a few realisations have been demonstrated [20,21]. However, none of these have been stabilized and positioned on the sensor chip.

Noticeable works have also been performed in the realisation of SFA cameras in the visible [22,23,24], and in the NIR [25], without combining visible and NIR filters on the same sensor chip.

SFA differs in constraints and use from CFA, and some effort has been put into the pre-processing of such data, such as demosaicing [26,27,28,29,30,31], deblurring and denoising of spectral data [32]. Considering the last case in particular, a lot of effort has been put into the strategy to recover colors or improving color images from the joint acquisition of color and NIR information [29,32,33,34,35,36,37,38]. In addition, camera sensitivities may be optimized. Indeed, one could consider to select the best filters for pre-processing purpose, such as energy balance to fit specific material or lighting conditions [39] and demosaicing [40]. Filters may be also optimized for a given application, from very general reflectance estimation [41] to specific analysis, i.e., medical imaging applications [42]. Although in theory and simulation good results can be achieved, the practical realization of filters and resulting camera sensitivities are usually not meeting very well the expectations. For instance, a Gaussian model was used in [40] to optimize the sensitivities for demosaicing, but selected curves are not quite similar to the resulting practical sensor that is shown in [22]. This is a material limitation to filter optimization so far, which will be overcome in the future development of transmittance filter technologies.

While Lapray et al. [17] focused on demonstrating the filter technology and on how to assemble the camera, in this paper we propose to perform the demonstration and spectral characterization of the whole SFA based MSI camera. Indeed, this prototype is designed to acquire multispectral data at single shot, based on a Complementary Metal Oxide Semi-conductor (CMOS) sensor and SFA filters. Both articles should be considered as complementary and not overlapping. Lapray et al. [17] focused on the state of the art of snapshot multispectral imaging and on the realisation and spectral analysis of the filter. They consider also how to combine them within a camera. This paper is an investigation on the realized camera, where we focus on the *final product* and before a specific application. We begin with the description of the hardware system and of its assembly in Section 2. Then, we apply denoising and pre-processing on the raw data. These raw data are used to measure the spectral characteristics of the SFA sensor in Section 3. In Section 4, we demonstrate the acquisition of real scene and show images based on demosaicing and color calibration. All these data are provided as a database for further studies, comparison and validation of simulation within the imaging and sensor communities.

## 2. Description of the System

SFA imaging technology is the central subject of this work. SFA is essentially a spatio-spectral sampling mechanism where the number of spectral bands may vary a lot both in number and shape. The choice of filters can be rather specific to the application. We consider here a general-purpose system that spans the visible and NIR part of the electromagnetic spectrum to serve as a proof of concept and further research. We propose a generic SFA arrangement with 8 channels. This section concerns the description of the system in term of hardware design.

### 2.1. Camera Architecture

**Figure 1:**
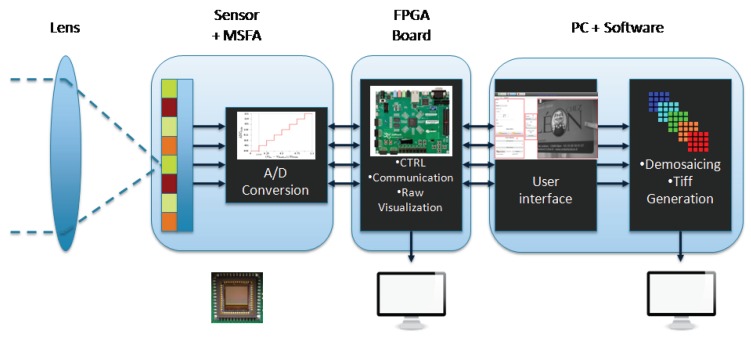
Overall architecture of the ad hoc imaging system. It is composed of four hardware blocks with dedicated features, through capture to processing. The video output can be visualized both before and after pre-processing, via respectively the FPGA board and the PC application.

The system is developed in order to acquire multispectral images and video sequences. The global pipeline is shown in Figure 1. The first element is a lens which focuses the incoming light onto the sensor plane. Then, the light is passing through a single monochrome image sensor (CMOS sensor Sapphire EV76C661 from E2V [43]), covered by a custom SFA layer aligned with the sensor pixels and stuck on it with a glue.

The image sensor offers a 10-bit digital readout speed at 60 frames per second (fps) with the global shutter method acquisition. The size of each pixel is 5.3 × 5.3 μm^2^, for a spatial resolution of 1280×1024 pixels. A Field Programmable Gate Array (FPGA) receives the digitized uncompressed data from the sensor, organizes it as a video stream and transmits it to a computer via Ethernet UDP protocol or directly to a monitor through an HDMI protocol. We can notice that this kind of architecture is suitable for an embedded camera architecture, which could provide intelligent processing inside like in Lapray et al. [44], for future development and applications. Finally, a software running on a PC is used to receive, demosaic and save the data coming from the camera for analysis purpose.

**Figure 2:**
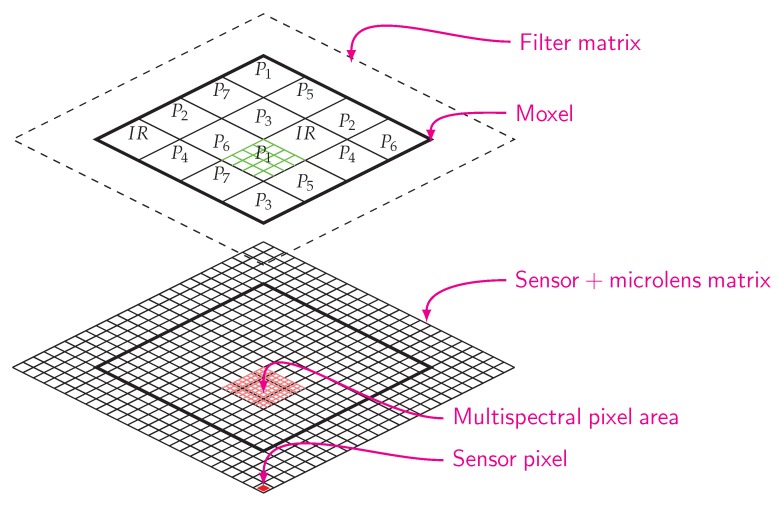
The mosaic arrangement is a moxel assembly over a monochrome sensor. The sensor matrix is composed of photodiodes and a microlens array. Each channel filter covers 4 × 4 sensor pixels.

Conventional sensors achieve color imaging by holding a CFA situated between the photodetectors and the microlens array. In our design, we do not remove the lens array before mounting the SFA, so that the filters are mounted over the original lens array attached to the sensor (see Figure 2). This process differs from the technique used in industry but has been used in a few works [20], however they did not fix the filters over the sensor.

**Figure 3:**
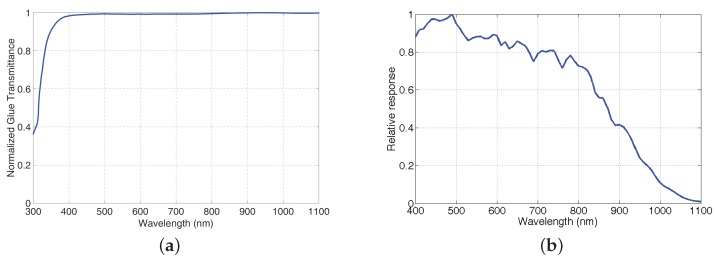
Glue transmittance (**a**) and sensor efficiency of the CMOS sensor EV76C661 (**b**).

The selected sensor provides a relatively good sensitivity in the NIR spectrum (quantum efficiency >50 % at 860 nm), while keeping good performance in the visible spectrum (about 80%). Due to the generally low transmission factors of the Fabry-Perrot filters, it is important to have a good sensor sensitivity in order to keep a low exposure time, thus to keep the maximum frame rate available for video purpose. The relative quantum efficiency of the nude sensor is shown on Figure 3b.

The customized matrix of filters is built by SILIOS technologies [45]. SILIOS Technologies developed the COLOR SHADES^®^ technology, allowing the manufacture of transmittance multispectral filters. COLOR SHADES^®^ technology is based on the combination of thin film deposition and micro-/nano-etching processes onto a fused silica substrate. Standard micro-photolithography steps are used to define the cell geometry of the multispectral filter. COLOR SHADES^®^ provides band pass filters originally in the visible range from 400 nm to 700 nm. Through our collaboration, SILIOS developed the filters in the NIR range, combining their technology with a classical thin layer interference technology to realize assembled filters. Filters transmittance have been extensively studied by Lapray et al. [17]. The SFA contains eight filters, referred to as {P1,P2,P3,P4,P5,P6,P7,IR}. Due to the constraints and difficulties to realize the filters in practice, we did not aim at optimizing their distribution for any specific application, but concentrated on having a balanced sensor with equidistant peaks, at least in the visible part that we controlled better.

### 2.2. Spatial Arrangement

Mosaic arrangement impacts directly on the image resolution through the demosaicing process. In a mosaic pattern, each pixel captures only one value relative to one spectral sensitivity at a time. Other spectral band values can be estimated using the neighboring pixels of a given band. The increasing number of spectral channels, increases the sparsity of channel occurrence and make the demosaicing more difficult than in the CFA case.

Miao et al. [46,47] proposed a generic mosaicing and demosaicing algorithm that is the most comprehensive definition in the literature. They take into account the probability of appearance of the channels, the spectral consistency and the uniformity of the distribution with their method. This implementation is based on the binary tree decomposition, given a number of spectral band and the probability of appearance of each band. They also propose a demosaicing technique, where the interpolation is done by order, with a first interpolation for the spectral band that has the highest probability of appearance.

**Figure 4:**
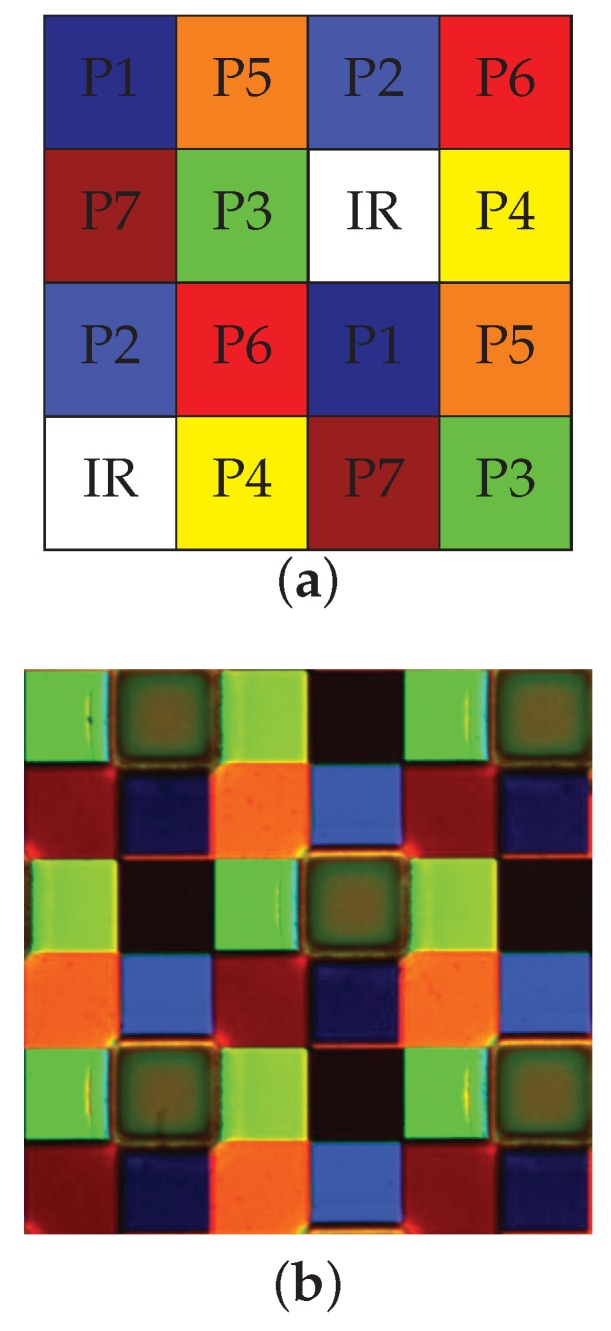
Visualization of moxel spatial arrangement. (**a**) Moxel arrangement for P1−P7,NIR channels. It shows uniformly distributed samples as an instance of Miao et al. [46,47] binary tree algorithm; (**b**) Microscope image of the MSFA filters after they are manufactured and before to mount them on the sensor.

According to their work, we define a periodic spatial distribution corresponding to this approach that promotes the spectral information recovery, i.e., each channel has the same probability of occurrence, 1/8. The filter arrangement chosen is shown in Figure 4a. A microscope image of this arrangement after manufacturing the filter is shown on Figure 4b.

The manufacturing process had required us to have 16 (4 × 4) adjacent photosensitive elements for one filter. So each color square has an area of 21.2 × 21.2 μm^2^ (4 × 5.3 × 5.3 μm^2^), with an uncertainty level. At the corners of the filter layer, some marks permit to identify pixel positions. The filters are then positioned over the sensor by active alignment where an image is recorded from the sensor and magnified: We could thus see when the filters are aligned in real time. Filters are glued on-top of the sensor in the same process. The transmittance of the glue (see in Figure 3a and its affect was not studied in the work of Lapray et al. The transmittance of the glue is consistent at ±2% between 400 and 1100 nm and is superior to 95% for the thickness used. It is interesting to notice that the glue acts as a UV-cut filter and limit the sensor noise that could come from UV radiations.

Once this is done, the sensor is combined with our camera system and can be used.

## 3. Spectral Characterization

This section considers the characterization of the spectral sensitivities of the sensor. To this aim, we propose a pre-processing of the measured data before to investigate the spatio-spectral properties of the sensor.

### 3.1. Pre-Processing

The pre-processing includes a dark correction to account for dark noise and a downsampling of the image to account for cross-talk, leakage and inaccuracy in filter realisation. This is made at the expense of the spatial resolution.

#### 3.1.1. Dark Master

At a specific integration time, we create a dark master, $I_{Dark}$, based on a set of N=10 images of dark $I_{d}^{n}$, with *n* an integer such as n∈[1,10]. For each pixel, we select the median values of the pixels from the set, such as in Equation (1).

(1) $$I_{Dark}\left(i,j\right)=median\left(I_{d}^{n}\left(i,j\right)\right)$$

The resulting image $I_{Dark}$ is subtracted from all images taken with this integration time. In the following, all images and measurements have been accordingly corrected. When the subtraction gives negative values, we are clipping them to 0. This dark image correction is standard and is described in several works [48,49].

#### 3.1.2. Downsampling

**Figure 5:**
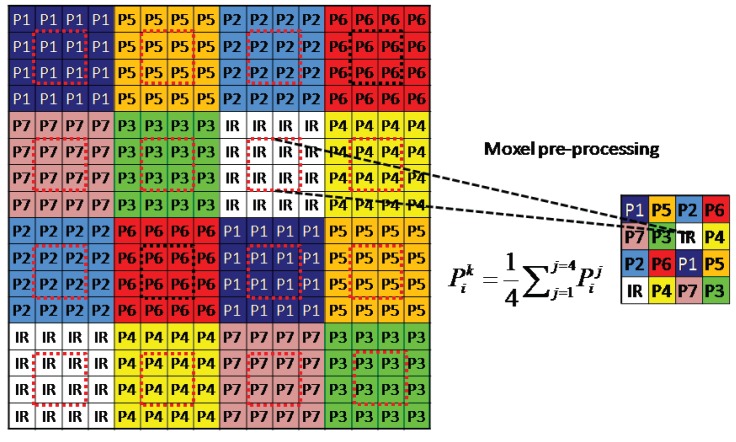
Pre-processing (downsampling) applied before using pixel values of images. We select the four center pixels to filter the spatial non-uniformity related to each spectral bands.

**Figure A1:**
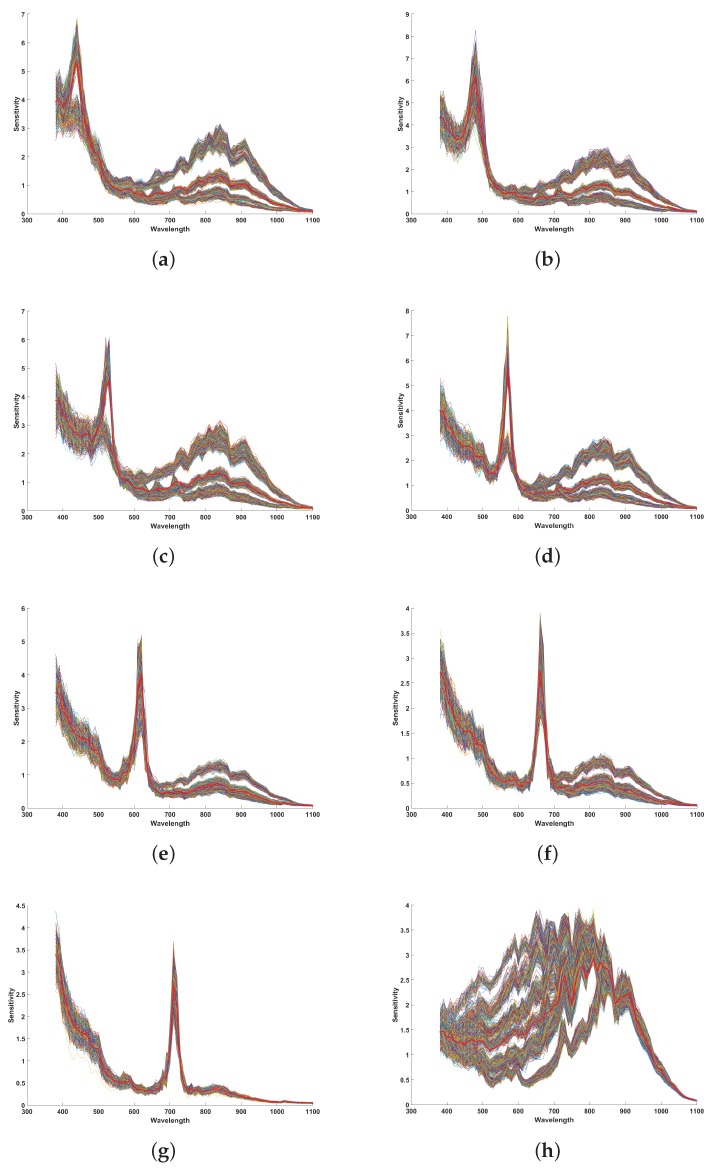
Spectral variation of camera sensitivities over sensor pixels, without applying any pre-processing on the raw images. In red, the average curve per band. (**a**) Sensitivity 1 over pixels; (**b**) Sensitivity 2 over pixels; (**c**) Sensitivity 3 over pixels; (**d**) Sensitivity 4 over pixels; (**e**) Sensitivity 5 over pixels; (**f**) Sensitivity 6 over pixels; (**g**) Sensitivity 7 over pixels; (**h**) Sensitivity 8 over pixels.

**Figure A2:**
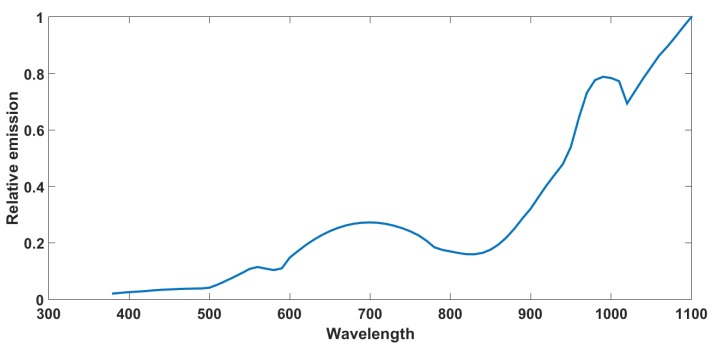
Relative energy emission by wavelength of the light source used in the measurements. The light source has been measured by using the *OL DH-300EC* silicon detector.

Figure 4b shows inaccuracy in filter realization and shows also that the NIR filter is overlapping on the connected cells. In addition, the filter layer is positioned at some distance of the micro-lenses, which creates cross-talk on neighbor pixels. Without any pre-processing, we observed that the bands of the visible domain transmit a part of the intensity range in the NIR. The shape of P1−P7 response curves seemed to be consistent with the infrared channel itself. In addition, the light that hit the bands P1, P2, P3 and P4 appeared to pass through the wavelength range of 780−1100 nm, and in a greater magnitude compared to bands P5 and P6. We also observed that P7 was very poorly affected by this phenomenon due to its position in the mosaic. This behavior is explained by the fact that the bands passing infrared light are located physically closer to the pixels of the infrared band. This effect highlights the technical difficulties in obtaining good filters and alignment, physically uncorrelated and without overlap between materials. In order to denoise these data, we decided to sacrifice the contiguous pixels, at the expense of the spatial resolution of our camera. As we can see in Figure 5, we take the four center pixels for each channel, and make the average of them to build a new downsampled image. The spatial resolution of the sensor becomes then 320×256 pixels. This pre-processing provided a noticeable improvement and confirmed our hypothesis on leakage, cross-talk and spatial filter pollution. Reader can refer to the Figure A1 and Figure A2 in the Appendix to see how the processing improved the quality of the sensor.

### 3.2. Spectral Sensitivities

#### 3.2.1. Spectral Characterization

We measure the relative spectral response of the camera system in a white room controlled environment. The measurement system is composed of a light source based on the halogen quartz lamp of the *OL 740-20D/UV Source Attachment* and of a double monochromator *OL 750-M-D Double Monochromator* that includes spherical mirrors to concentrate and collimate the light. Both are from http://goochandhousego.com/Gooch & Housego Company.

We sweep the wavelength of the incoming light by step of 10 nm from 380 nm to 1100 nm. We capture a picture for each wavelength with an integration time of 0.503 ms, which permits no saturation of any of the channels but maximizes the incoming signal to limit noise. We repeat the procedure for two sets of captures in order to minimize error in measurements. These two sets are averaged after the pre-processing is applied.

For the integration time, the actions for the calibration of the camera are:

1. Create a Dark Master image for the given exposure time as described in Section 3.1.1.
2. Downsample and pre-process the images as described in Section 3.1.1 and Section 3.1.2.
3. Capture 2 sets of images of monochromatic light.
4. Average the 2 image sets.
5. Select a square of 84 pixels at the center of each image, where a small angle inaccuracy would be negligible and where the monochromatic light is assumed to be uniform according to the specification of our devices, with a large security margin.
6. Sort out pixels by filter type and apply light source monochromator calibration to the data.
7. Average the curves from the 84 × 84 pixels.
8. Normalize the curve over the highest number. By doing that, we preserve the ratio of efficiencies by channel, assuming a linear sensor.

**Figure 6:**
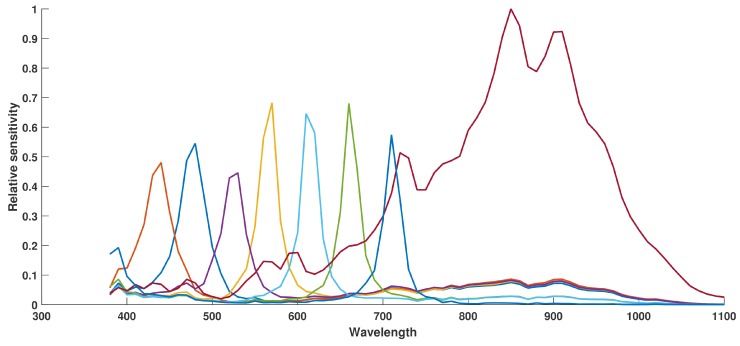
The actual SFA MSI system relative spectral sensitivities after pre-processing, as described in Section 3.1. This is the measure of the relative efficiencies of each channel, and no simulation is added. Curves data are provided as Supplementary Materials in file Excel S1.

Finally, by using this technique of calibration, the curves of the effective camera response with filters are shown on Figure 6.

#### 3.2.2. Analysis

We study the spectral interference between camera sensitivities by spectral bands and the spatial uniformity over the sensor.

We compute the interference values, Θ, between each spectral profile pairs in Table 1. The mutual interferences are quantified, according to previous works [50] and extended from filters to sensitivities (We modified the integral boundaries so we include the multi-modalities of the sensitivities in the evaluation.), by determining the interference coefficients computed by using the ratio between overlapping area of two sensitivities over one of these sensitivities, as in Equation (2):

(2) $$\Theta =\frac{\int_{380}^{\lambda c}S_{i}\left(\lambda \right).d\lambda +\int_{\lambda c}^{1100}S_{j}\left(\lambda \right).d\lambda }{\int_{380}^{1100}S_{j}\left(\lambda \right).d\lambda }$$

where 380 and 1100 are limits of the wavelength interval of interest and λc is the wavelength at the intersection of $S_{i}$ and $S_{j}$. λc is evaluated manually from the curves in Figure 6.

We observe that in the visible, band P7 has a harmonic transmittance peak that lets pass light up to 400 nm, this is a typical Farby-Perrot behavior. This increases correlation between P1 and P7. Rest of the visible bands show an expected correlation. Despite of the pre-processing, there is still cross-talk and leakage in the NIR to be noticed on bands P1−P4. The NIR channel shows a noticeable sensitivity in the visible, which is a critical limitation for applications in computer vision, which would benefit from a good separability between visible and NIR. This is inherently a problem of a bad control in filter realization. These leakage and visible-NIR pollution may be handled and corrected with a post-processing similar to what is done in works such as in Sadeghipoor et al. [29].

**Figure 7:**
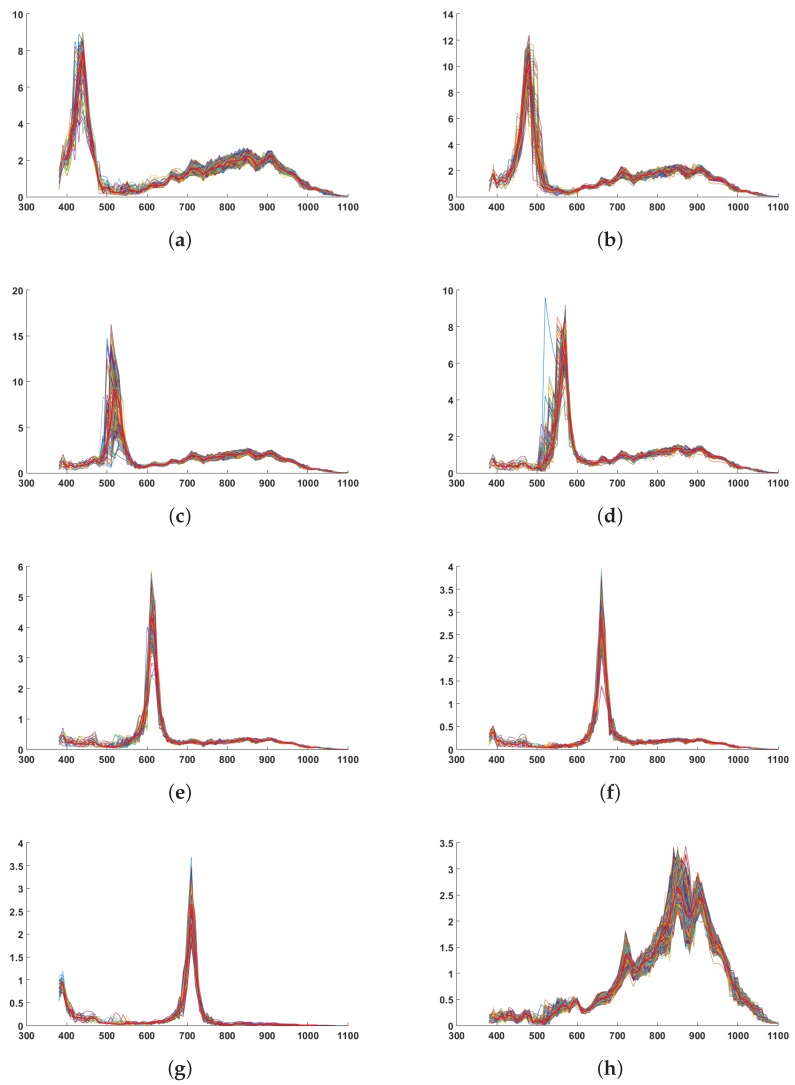
Spectral variation of sensor sensitivities along with the spatial dimension. In red, the average curve. Relatively to the pixel response, some of the bands have a more or less good rejection in the IR wavelengths. It seems to be directly correlated to the adjacency of the filter with the IR pixels in the moxel arrangement. (**a**) Sensitivity 1 over pixels; (**b**) Sensitivity 2 over pixels; (**c**) Sensitivity 3 over pixels; (**d**) Sensitivity 4 over pixels; (**e**) Sensitivity 5 over pixels; (**f**) Sensitivity 6 over pixels; (**g**) Sensitivity 7 over pixels; (**h**) Sensitivity 8 over pixels.

We also investigate qualitatively the spatial uniformity of the filters over our square of 84 pixels. Results are shown in Figure 7, where we plot the sensitivities for all the pixels within the center window of 84×84 by spectral bands. We observe a reasonably good consistency for a single prototype.

A quantitative analysis is performed by analyzing the average of the variances, computed at each point of these curves, every 10 nm, around the average curve. Results are shown in Table 2. It seems that filters closer to the NIR filter are showing more variance, which may be explained by some leakage as we discussed in the pre-processing step. We observe also that the bands that are showing a larger variance have the peak sensitivity which is shift on Figure 7. This is not easy to explain in an affirmative way since this may be related to the measurement sampling every 10 nm, or to the technology instability in the filter realization.

In addition, the quantitative impact of the variance in sensitivities on the multispectral image quality is yet to be analyzed. In general, both the drawbacks of spatial uniformity and quality in filter realization will be overcome to some extend with the development of the technology and with the industrialization of the process.

## 4. Multispectral Imaging

In this section we demonstrate the capability of the sensor to capture multispectral images.

### 4.1. Energy Balance

Energy balance is important for single sensor spectral imaging [39] in order to minimize the noise and balance it between channels. Indeed, it might happen that one channel get saturated while another does not get enough incoming light, which would impair critically the application.

**Figure 8:**
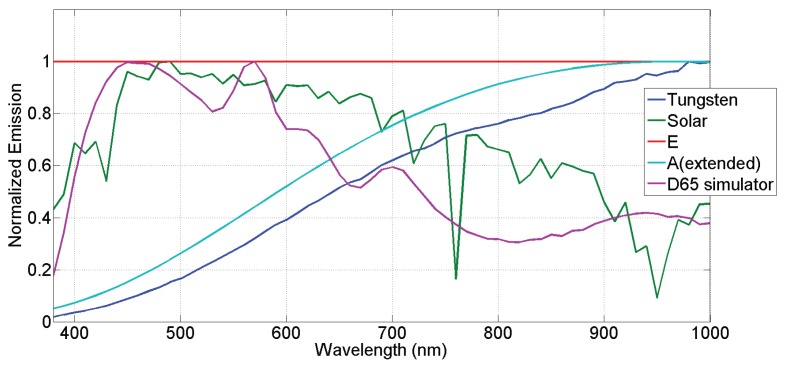
Multiple illuminants used for the energy balance study given in Table 3. The tested wavelength range is 380–1000 nm. The D65 simulator spectral emission is used for the acquisition of the image database. Illuminant data are provided as Supplementary Materials in file Excel S2.

In Table 3, we show the relative acquisition value for a perfect diffuser enlightened with different illuminations. For each tested illuminant shown in Figure 8, the results are normalized by the maximum value of the visible bands. We call $\rho_{p}$ the response of the camera according to a simple model of image formation assuming the perfect diffuser reflectance, such as defined in Equation (3):

(3) $$\rho_{p}=\int_{\lambda min}^{\lambda max}I\left(\lambda \right).S_{p}\left(\lambda \right).d\lambda$$

where I(λ) is the spectral emission of the tested illuminant, S(λ) is the camera response shown in Figure 6 and *p* the index of the spectral band.

The Commission Internationale de l’Eclairage (CIE) standard illuminants cannot be used here, as we consider also the NIR part of the spectrum, that is not described yet by these standards. Thus, we selected and computed alternative illuminations. We selected a measure of solar emission at the ground level, performed a measure of a D65 simulator, we computed the theoretical black body emission (A illuminant) and use a measure of its practical tungsten realization. In addition, we used also illuminant E as a reference.

We note that the energetic distribution is reasonably well balanced in the visible range with natural exposures, since the variance between the spectral bands is acceptable, according to typical RGB cameras, for all tested illuminants. All these results can be indeed compared to the typical curves of the RGB Sinarback camera [51], where the camera response variance is considered to be good enough for the sensor energy balance of an RGB device. When it comes to the joint acquisition of visible and NIR, we note the quite large difference between values of *ρ*. Such acquisition may benefit from a similar-to-HDR acquisition process, but this would require multiple captures with different integration times. However, at the expense of noise, we experienced the feasibility of the set-up.

### 4.2. Images Acquired

**Figure 9:**
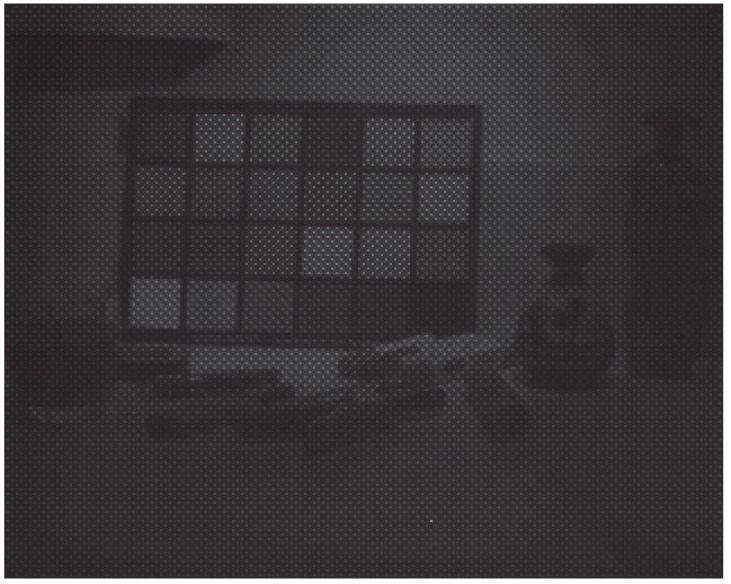
Raw image of scene 1 after applying the pre-processing described in Section 3.1. The integration time was of 16.082 ms. RAWimage is provided as Supplementary Materials in Figure S1.

**Figure 10:**
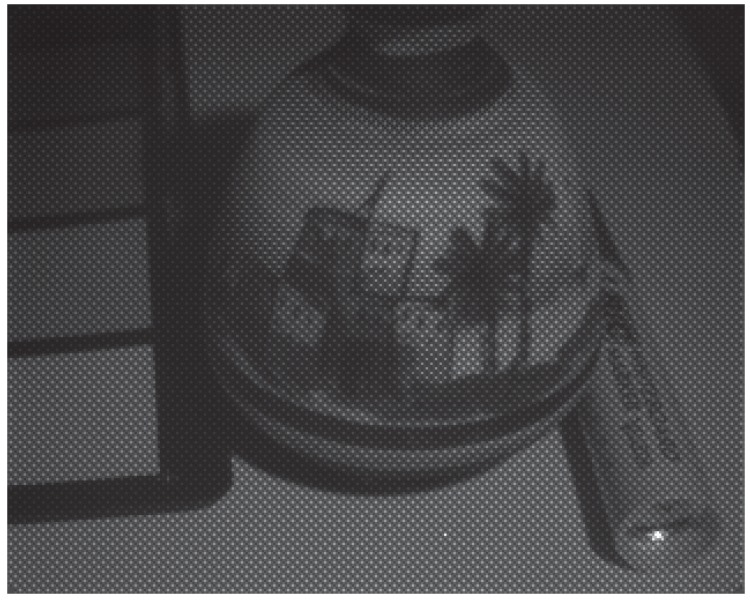
Raw image of scene 2 after applying the pre-processing described in Section 3.1. The integration time was of 8.049 ms. RAWimage is provided as Supplementary Materials in Figure S2.

Images were captured in order to illustrate the practical results of our system. The images have been taken under the D65 simulator, which relative spectral emission is shown in Figure 8. This illuminant proved to be not very well spatially uniform, but we implemented no flat field corrections, so spatial non-uniformity in the pictures may be due to effect of lenses and to effect of illumination. The Figure 9 and Figure 10 show two examples of a mosaiced raw image of two scenes captured with the camera. By zooming in the pixel pattern, we can clearly distinguish the moxel arrangement defined in Figure 4a.

**Figure 11:**
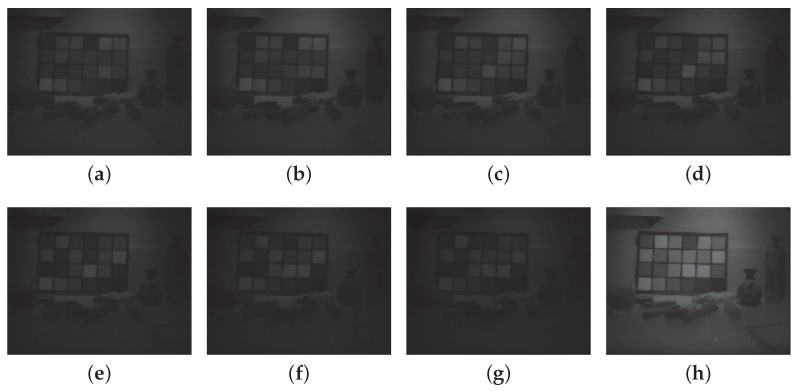
Demosaiced image by Miao binary tree algorithm. Channels 1 to 8 are reconstructed to provide a full resolution multispectral image. These data are encapsulated in one single tiff file for storage in Supplementary Materials. (**a**) Channel P1; (**b**) Channel P2; (**c**) Channel P3; (**d**) Channel P4; (**e**) Channel P5; (**f**) Channel P6; (**g**) Channel P7; (**h**) Channel IR. Demosaiced image is provided as a tiff file as Supplementary Materials in Figure S3.

**Figure 12:**
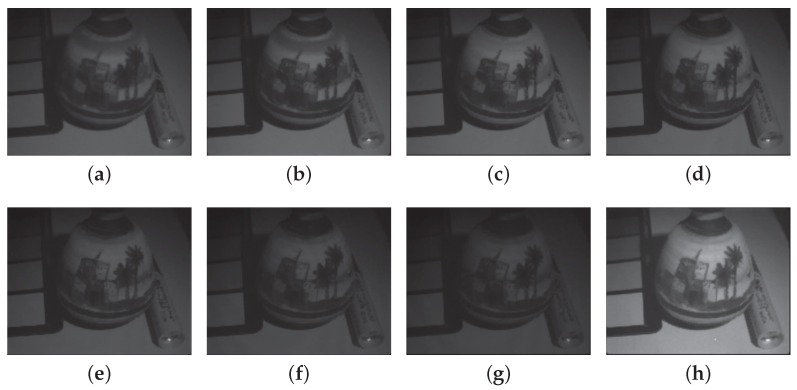
Demosaiced image by Miao binary tree algorithm. Channels 1 to 8 are reconstructed to provide a full resolution multispectral image. These data are encapsulated in one single tiff file for storage in Supplementary Materials. (**a**) Channel P1; (**b**) Channel P2; (**c**) Channel P3; (**d**) Channel P4; (**e**) Channel P5; (**f**) Channel P6; (**g**) Channel P7; (**h**) Channel IR. Demosaiced image is provided as a tiff file as Supplementary Materials in Figure S4.

Images were demosaiced with Miao et al. [46,47] binary tree algorithm for benchmark. Figure 11 and Figure 12 show the 8 bands reconstructed by the demosaicing process for scene 1 and 2.

**Figure 13:**
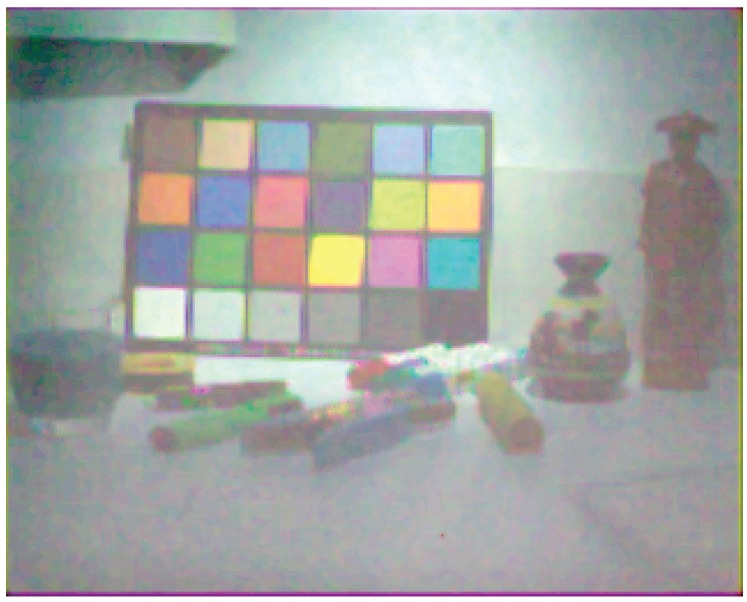
sRGB image of scene 1 computed according to a colorimetric calibration of the sensor. Color image is provided as Supplementary Materials in Figure S5.

**Figure 14:**
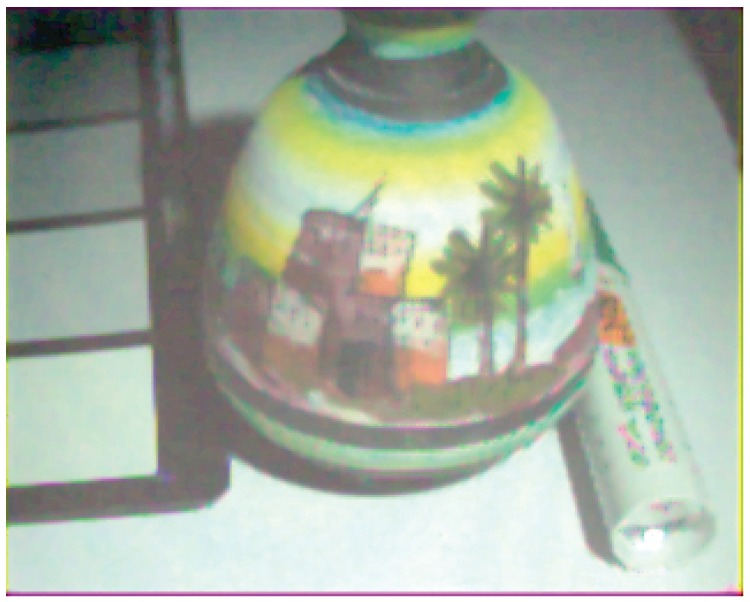
sRGB image of scene 2 computed according to a colorimetric calibration of the sensor. Color image is provided as Supplementary Materials in Figure S6.

It may be easier to visualize the data as color images. For color visualization of the images, we performed a colorimetric calibration of the sensor based on the Gretag MacBeth color checker measured reflectances between 380 and 1100 nm. We used the 1931 2° CIE observer and adequate CIE computations to compute the tristimulus values at every pixel, and then computed sRGB data. The model is a straightforward linear model which compute XYZ values from the 8 sensor values. The illuminant data used is the D65 simulator. The color image of the scenes are shown in Figure 13 and Figure 14.

## 5. Conclusions

In this work, we presented and characterized a multispectral camera based on SFA. Our contribution lies in the practical study of a SFA design and its real implementation.

This system shows some advantages compared to existing multispectral capture: it has an exact registration of the images, it can be low cost compared to actual MSI systems, with some compactness and robustness advantages, and it opens opportunities for on-the-fly analysis and video processing. This kind of design could be suitable for many types of CMOS/CCD sensor applications, regardless of the resolution, the frame rate or the implemented pixel technology.

However, the technique of mounting filters directly on the lens array can increase the amount of optical crosstalk. Indeed, the filter array is spaced relatively far from the lens array (in part due to the glue), a photon interacts firstly with the incoming matrix filters before to interact with the lens array. And future directions of work include to build a setup that leads to amount filters directly on the sensor.

We demonstrated the feasibility of the system and developed it up to color image representation. We provide our experimental data and a couple of acquired scenes to the community. The provided data and images may be used to benchmark methods and algorithms related to multispectral image acquisition, surface object properties estimation (reflectance reconstruction) and demosaicing as well as denoising and image restoration. Data may also be used for pseudo-real simulation with a ground base.

Designing optimal camera peak sensitivities for specific applications become possible now that we can provide a demonstration of the realization in practice of a working system. This may lead to interesting development of the technology in the future and new methodologies to tackle open problems. In addition, further work on deblurring and other optical corrections may be considered as well as significant contributions in optimizing the filters for specific application or energy balance of the sensor.

## Figures and Tables

**Table 1:** Mutual spectral interference Θ coefficients of the spectral sensor efficiencies.

i ∖ j	P1	P2	P3	P4	P5	P6	P7	IR
**P1**	1.0000	0.7131	0.6331	0.5745	0.8218	0.8472	1.1280	0.1352
**P2**	0.7162	1.0000	0.6534	0.5363	0.7506	0.7691	1.0292	0.1252
**P3**	0.6161	0.6332	1.0000	0.6688	0.8517	0.8522	1.1084	0.1554
**P4**	0.5853	0.5440	0.7001	1.0000	0.8865	0.8245	1.0676	0.1632
**P5**	0.5737	0.5218	0.6110	0.6075	1.0000	0.4891	0.6001	0.1798
**P6**	0.5808	0.5250	0.6003	0.5548	0.4802	1.0000	0.6869	0.2018
**P7**	0.6261	0.5688	0.6321	0.5817	0.4771	0.5562	1.0000	0.2090
**IR**	1.0290	0.9667	1.0495	0.9707	0.9999	1.2021	1.7978	1.0000

**Table 2:** For each spectral band, we show the average of the variances, computed at each point of these curves, around the average curve.

Bands	P1	P2	P3	P4	P5	P6	P7	IR
**Average variances**	0.0943	0.3585	0.4681	0.0666	0.0215	0.0089	0.0105	0.0198

**Table 3:** Relative values of the sensor response by the filter ($\rho_{p}$), for a given input illuminant ($I_{\lambda }$) and a perfect diffuser. Illuminant E is extended to the NIR and the simulator of D65 used in image acquisition has been measured up to 1000 nm. All the illuminant emissions are visible in Figure 8.

Illuminant	E	Tungsten	D65 Simu.	A (Extended)	Solar
$R_{Sinarback}$	0.47	0.70	0.40	0.66	0.45
$G_{Sinarback}$	1	1	1	1	1
$B_{Sinarback}$	0.82	0.46	0.85	0.50	0.79
$P_{1}$	0.97	0.62	0.93	0.66	0.87
$P_{2}$	0.98	0.69	0.99	0.73	0.99
$P_{3}$	0.88	0.79	0.81	0.82	0.87
$P_{4}$	1	0.95	1	0.98	1
$P_{5}$	0.87	0.88	0.79	0.90	0.88
$P_{6}$	0.85	1	0.63	1	0.82
$P_{7}$	0.73	0.85	0.55	0.84	0.64
IR (380–780 nm)	2.06	2.77	1.53	2.71	1.83
IR (380–1000 nm)	4.21	5.84	2.87	5.55	3.34

## Supplementary Materials

The following are available online at www.mdpi.com/1424-8220/16/7/993/s1, Excel S1: SensorSensitivities; Excel S2: IlluminantData; Figure S1: Scene1-D65-raw_8_bits-16-082-Preprocessed; Figure S2: Scene2-D65-raw_8_bits-8-049-Preprocessed; Figure S3: Demosaiced-binaryTree-Preprocessed-Scene1-D65-raw_8_bits-16-082; Figure S4: Demosaiced-binaryTree-Preprocessed-Scene1-D65-raw_8_bits-8-049; Figure S5: Scene1-D65-color_8_bits-16-082-Preprocessed; Figure S6: Scene2-D65-color_8_bits-8-049-Preprocessed.

## Appendix A

This appendix proposes a deeper insight into the effect of the pre-processing we propose. It serves also as a qualitative evaluation and justification of this process. Raw images are pre-processed in our camera framework to make a better image by taking into account a few observations. These observations are discussed in the following.

Figure A1 shows the sensitivities of each pixel of the sensor sorted by type of filters that cover them. These are data without any processing. From these curves, we make three observations.

1. We observe a large offset common to all the channels in shorter wavelengths.
2. We observe a large bandpass behavior in the NIR for the bands that have peaks in the visible (Figure A1a,g). We observe that the behavior of some pixels are quite different in this part.
3. We observe that the channel that have peak in the NIR (Figure A1h) has a huge variance compared to visible spectral bands.

Thus, we provide the following analysis:

1. The large offset common to all channels is higher for the short wavelengths. This is related and proportional to the weakness of the light source used in the experiment, shown in Figure A2, in this range of wavelengths. We argue this would be a multiplicative contribution of the dark noise while accounting for the energy of the light source. This can be corrected by applying a dark noise correction to all acquired images.
2. According to the specific behavior in the NIR sensitivity of the visible bands, we argue that this is directly related to the proximity of an infrared pixel. This is induced by an inaccuracy in NIR filter realization as can be seen in Figure 4b. The NIR filter is overlapping on the connected cells. In addition, the filter layer is positioned at some distance of the micro-lenses, which creates cross-talk on neighbor pixels. Without any pre-processing, we observed that the bands of the visible domain transmit a part of the intensity range in the NIR. The shape of P1−P7 (Figure A1a,g) response curves seemed to be rather consistent with the NIR channel itself. In addition, the light that hits the bands P1, P2, P3 and P4 appeared to pass through the wavelength range of 780–1100 nm, and in a greater magnitude compared to bands P5 and P6. We also observed that P7 was very poorly affected by this phenomenon due to its position in the mosaic. This behavior is explained by the fact that the bands located physically closer to the pixels of the NIR band are affected by it. This effect can be partially corrected by selecting pixels at the center of each filters and discarding the others.
3. The big variance in the NIR channel is due to the process of thin layer deposition, which is different from the micro-/nano-etching of the visible bands. Different behaviors can be observed sliding from a high sensitivity in the visible to less. This behavior resembles variation of transmittance filters according to a gradient thin layer deposition. Indeed, we could explain this behavior by a graduated thickness of the NIR filter. This effect can be partially corrected by selecting the pixels at the center of the NIR filters, where the thin layer is supposed flat and uniform and discarding the others.

Pre-processings include a dark correction to account for dark noise, and a downsampling to account for cross-talk, leakage and inaccuracy in filter realization. This is made at the expense of the spatial resolution. Benefits can be clearly observed by looking at Figure 7. This result confirms our observations.
