# Supplementary figures and images for: Spectral Characterization of a Prototype SFA Camera for Joint Visible and NIR Acquisition
Source: Sensors (Basel). 2016 Jun 28;16(7):993. doi: 10.3390/s16070993 (PMC4969836; doi:10.3390/s16070993)

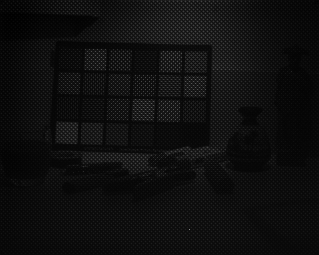

Supplement: Supplementary file 1 [file sensors-16-00993-s001.zip › Figure S1_Scene1-D65-raw_8_bits-16-082-Preprocessed.tiff]

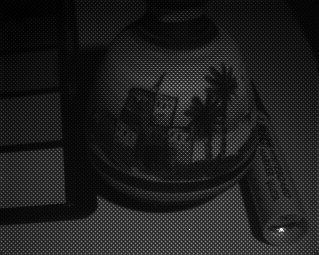

Supplement: Supplementary file 1 [file sensors-16-00993-s001.zip › Figure S2_Scene2-D65-raw_8_bits-8-049-Preprocessed.tiff]

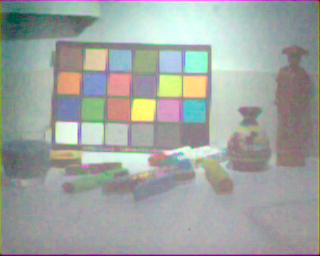

Supplement: Supplementary file 1 [file sensors-16-00993-s001.zip › Figure S5_Scene1-D65-color_8_bits-16-082-Preprocessed.png]

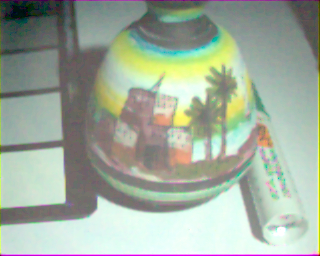

Supplement: Supplementary file 1 [file sensors-16-00993-s001.zip › Figure S6_Scene2-D65-color_8_bits-8-049-Preprocessed.png]
